# Delivering intrapulmonary percussive ventilation physiotherapy with assisted autogenic drainage is associated with a lower incidence of ventilator-associated gram-negative infection

**DOI:** 10.1186/2197-425X-3-S1-A443

**Published:** 2015-10-01

**Authors:** M Borremans, M Diltoer, J De Regt, PM Honoré, C Bruggemans, H Spapen

**Affiliations:** University Hospital Brussels, ICU, Brussels, Belgium

## Introduction

Chest physiotherapy is frequently provided to ICU patients but its role remains questionable. Intrapulmonary percussive ventilation physiotherapy with assisted autogenic drainage (IPV-AAD) is an emerging form of chest physiotherapy designed to enhance clearance of endobronchial secretions whilst enhancing alveolar recruitment.

## Objectives

To evaluate the effect of IPV-AAD on the occurrence of Gram-negative (GN) ventilator-associated tracheobronchitis (VAT) or pneumonia (VAP).

## Methods

Consecutive patients admitted to a mixed medicosurgical ICU were eligible for study enrolment if they required oral intubation and mechanical ventilation for at least 48h. Patients who at outset presented any pulmonary infiltrate(s) were excluded. Patients were randomly assigned to receive either IPV-AAD, conventional physiotherapy (CPT) or no physiotherapy (NPT). CPT consisted of expiratory chest wall vibrations, positioning, rib-springing, aerosol therapy, and airway suctioning. The NPT group underwent mobilization, aerosol therapy and tracheobronchial aspiration. Standard institutional VAP precautions were guaranteed in all patients. IPV-AAD and CPT were performed by 2 dedicated and skilled respiratory therapists. Sessions were delivered twice daily for 20 min on a 24/7 basis. VAT and VAP were diagnosed according to established criteria (1). Study endpoint was clinically documented VAT or VAP plus a quantitative bacterial culture of at least 10^6^ CFU/mL of a true GN pathogen from an endotracheal specimen or at least 10^4^ CFU/mL from bronchoalveolar lavage fluid. Statistical analysis used non-parametric tests for independent samples and Fisher exact test to compare treatment groups.

## Results

Forty-five patients (24 males, 21females) were enrolled with 15 subjects included in each study arm. IPV-AAD patients were younger (46 ± 17 years) than CPT (62 ± 18 years) and NPT (64 ± 16 years) subjects (p= 0.014; IPV-AAD vs. CPT and NPT) but APACHE II scores were comparable between groups (20 ± 8, 23 ± 10 and 21 ± 6 respectively for IPV-AAD, CPT and NPT subjects, p= NS). GN VAT or VAP was diagnosed in 2 patients (13%) in the IPV-AAD group, 7 patients (47%) in the CPT group and 7 patients (47%) in the NPT group (p= 0.10; IPV-AAD vs. CPT and NPT). Time from start of the study till VAT/VAP diagnosis ranged from 3 to 11 days. Survival was not different between groups.

## Conclusions

In this small patient cohort, adjunctive IPV-AAD tended to decrease the incidence of ventilator-associated GN infection as compared with conventional or no chest physiotherapy.
